# Oral immunotherapy for peanut allergy: The con argument

**DOI:** 10.1016/j.waojou.2020.100445

**Published:** 2020-09-18

**Authors:** Alessandro Fiocchi, Maria Cristina Artesani, Vincenzo Fierro, Carla Riccardi, Lamia Dahdah, Maurizio Mennini

**Affiliations:** Allergy Department, Bambino Gesù Children's Hospital IRCCS, Rome, Italy

**Keywords:** Oral immunotherapy, Peanut allergy, Efficacy, Safety, Quality of life, EAACI, European academy of allergy asthma and immunology, ICER, Institute for clinical and economic review, OFC, Oral food challenge, OIT, Oral ImmunoTherapy, OUtMATCH, Omalizumab as monotherapy and as adjunct therapy to multi-allergen OIT in Food allergic participants, PACE, Peanut allergen immunotherapy, clarifying the evidence meta-analysis, POISED, Peanut oral immunotherapy Study:Safety, efficacy and discovery, POIT, Peanut oral ImmunoTherapy, QoL, Quality of life, SCIT, Subcutaneous immunotherapy, SLIT, Sublingual immunotherapy, SPT, Skin prick test

## Abstract

**Background:**

In some countries of the world, peanut allergy represents an important source of anaphylactic reactions. Traditionally treated with the avoidance of responsible allergens, this condition can also be targeted by oral peanut immunotherapy.

**Methods:**

In this study, we review the beneficial and side effects of currently available forms of peanut oral immunotherapy (POIT). We report the discussions resulting from the publication of a meta-analysis that brought to light the downsides of oral immunotherapy for peanuts.

**Results:**

In some clinical situations, the risk-benefit ratio can favor peanut oral immunotherapy over avoidance. In many other situations, this is not the case. The decision must be based on the values and preferences of clinicians and patients. Those not ready to accept serious adverse effects from POIT are likely to continue the elimination diet; those motivated to achieving desensitization, and prepared to accept serious adverse effects, may choose to undergo POIT.

**Conclusions:**

Without being prejudiced against peanut oral immunotherapy, we indicate the possible evolution of treatment for this condition is in a rapidly evolving broader scenario. Among the future options, sublingual immunotherapy, parenteral immunotherapy with modified allergens, transcutaneous immunotherapy, and the use of biologics will become important options.

## Introduction

Is it possible for a pediatric allergist to be against oral immunotherapy for peanut allergy? Certainly not. Our statutory goal is to treat the allergic disease, and the best treatment has consisted of desensitization for more than 100 years. It therefore would be unnatural for an allergist, especially a pediatrician, to be prejudicially opposed to oral immunotherapy for peanut allergy.

## The context

Peanut allergy, often characterized by severe reactions, has been reported to have increased in some parts of the world. In the UK, its prevalence rose from 500 to 1000 cases per 100 000 in 2 sequential cohorts born in 1989 and 1995–1997 in the Isle of Wight.^1^^,^^2^ This pathology is particularly present in preschoolers, with 105 cases per 100,000 in aged 0–4 years versus 13.4 per 100,000 in patients aged 5+ years.^3^

In the United States, data from surveys indicate that peanut allergy affects 1.8% of adults^4^ and 2.1 of children, with a reported increase between 1997 and 2008 in youngsters.^5^ Thus, the perception of an increase in peanut allergy in that country is extant.^6^

For some reason, peanut allergy is not as common in other European countries or in Asia and China. In Europe, the lifetime prevalence of self-reported peanut allergy was 0.4%, higher in adults and in Western Europe compared to children and Northern Europe.^7^ In Singapore, birth cohort data indicate the prevalence of peanut allergy at 0.1–0.3% in children aged 1–4 years.^8^ Data from hospital registers or surveys indicate that peanut allergy is prevalent in Hong Kong and Taiwan, but not in mainland China; in South Korea (estimated prevalence 0.22%) and the Philippines (0.43%), but not in Indonesia, Malaysia, Pakistan, Vietnam, and Thailand.^9^ Data on the prevalence of challenge confirmed peanut allergy in open populations are lacking in the majority of the world's countries.

Pediatric age is where the condition seems most malleable. In fact, the greatest frequency of spontaneous remission of the disease occurs in preschool children,^10^ and the early exposure to peanut proteins is able to prevent peanut allergy in infants suffering from eczema and egg allergy.^11^ Studies conducted on a peanut allergy population document that the effectiveness of immunotherapy is higher in children than in adults.^12^

## Peanut oral immunotherapy (OIT) can lead to desensitization, rarely to sustained unresponsiveness

For decades, the management of peanut allergy relied on avoidance strategies: patients should avoid contact and, above all, ingestion of this food. However, since the 1990s, there has been a need for more proactive treatment of food allergies. Already 100 years ago, one of the first allergy treatment experiments targeted egg allergy.^13^ Immunotherapy for food allergy is therefore as old as that for respiratory allergy. Italian authors published uncontrolled studies focused on early childhood allergens such as milk and egg.^14^^,^^15^ Since the late 1990s, the production of controlled studies by European and American authors has brought immunotherapy for food allergy from the pioneering to the scientific phase.

From this part of the Ocean, the enthusiasm for desensitization for milk and egg was dampened by the comparison between the results obtainable with OIT compared to the simple elimination diet.^16^ In America, the interest immediately focused on peanut, perceived as the food allergen of greater danger and prevalence. Initially, injective desensitization methods in use for respiratory allergies were applied to this type of allergy. It took little to understand that heavily modified extracts were needed to make this therapy safe.^17^ Subsequently, a series of studies on immunotherapy with oral peanuts showed conclusively that peanut OIT (POIT) is able to increase the threshold of reactivity to peanut proteins.^18^ In this way, it reduces the risk of reactions to the ingestion of small quantities of food, especially in traces. This effect is largely dependent on the continuation of therapy, and on its suspension tends to disappear. POIT therefore rarely gets “sustained unresponsiveness”,^19^ more often a desensitization only — ie, children “tolerate more food on treatment than before starting until they are treated”.^21^ Upon stopping treatment, they tend to become sensitive again, and its effect wears off.

On this ground, the guidelines of the European Academy of Allergy and Clinical Immunology (EAACI) recommended POIT “as a treatment option to increase the threshold of reaction while on treatment in children with peanut allergy from around 4–5 years of age”, while it was not recommended “as a treatment option to achieve post-discontinuation effectiveness”.^20^ Interestingly, a recent study confirms that discontinuation or reduction of the daily dose of POIT increases the likelihood of regaining clinical reactivity to peanut.^21^ This study indicates also that lower basophil activation at baseline predicts sustained unresponsiveness after 2 years, paving the way for predictive criteria for the effectiveness of POIT in particular phenotypes of peanut-allergic children.

In summary, we have known for at least 2 years the potential and limits of the effectiveness of peanut-specific OIT. However, a debate has developed over the past year on the tolerability of the procedure.

## The price for peanut desensitization using current approaches

From the metanalyses, the tolerability of POIT emerged as rather poor,^20^ so that and the risk/benefit balance should be carefully weighted. In this light, the PACE meta-analysis has measured the feasibility of POIT in the published literature.^22^ This study was conceived after the observation that the number of children treated with POIT in the published studies had reached a corpus as significant as to allow to completely reviewing the different aspects of the procedure. In the PACE metanalysis, twelve trials were included for a total of 1041 patients aged 5 to 14.8 years^14^^,^^23^, ^24^, ^25^, ^26^, ^27^, ^28^, ^29^, ^30^, ^31^, ^32^, ^33^^.^ The novelty of the approach consists of adopting as the main outcome the same used for immunotherapy for respiratory allergy, which is the symptomatology of food allergy outside the oral provocation test. This approach is also the one suggested by the US Food and Drug Administration (FDA), which indicated it as the most suitable to verify the efficacy and safety of food allergy treatments.^34^ In this perspective, the acquisition of desensitization witnessed by the diagnostic oral food challenge (OFC), as well as the increase of peanut protein thresholds at OFC, become a surrogate outcome. On the other hand, anaphylactic reactions and the need for use of epinephrine are particularly important, because they are technically definable as serious effects: they force an action to prevent a life-threatening state or hospitalization (Table 1).^35^ Another objective of interest is the need to stop the desensitizing treatment. In this way, the direct measures of POIT tolerability are the following:

a. Peanut-induced anaphylaxis: With 222 events per 1000 patients, compared to 71 events per 1000 patients, POIT results in a large increase in anaphylactic reactions compared to allergen avoidance or placebo. The relative effect is 3.12 (95%CI 1.76–5.55).
b. Epinephrine use: With 82 events per 1000 patients, compared to 37 events per 1000 patients on allergen avoidance or placebo, POIT results in an increase in rescue epinephrine use. The relative effect is 2.21 (95%CI 1.27–3.83).
c. Serious adverse events: With 62 events per 1000 patients, compared to 19 events per 1000 patients, POIT increases life-threatening reactions, or reactions severe enough to require urgent medical intervention or hospitalization to prevent this, compared to placebo. The relative effect is 1.92 (95%CI 1–3.66).
d. Quality of life: The likelihood of improving quality of life by POIT was nonsignificant (risk ratio to achieve minimally important difference: 1.14 [0.66–1.99] in parent-reported QoL, 1.20 [0.80–1.81] in self-reported QoL.
e. Treatment discontinuation: With 61 events per 1000 patients, compared to 24 events per 1000 patients, POIT increases reactions severe enough to drop out of a research study compared to placebo. The relative effect is 2.55 (95%CI 1.20–5.42).
f. Abdominal pain: With 463 events per 1000 patients, compared to 245 events per 1000 patients, POIT increases it compared to placebo. The relative effect is 1.89 (95%CI 1.45–2.46).
g. Any allergic reaction: With 119 events per 1000 patients, compared to 159 events per 1000 patients, POIT increases adverse reactions compared to placebo by a relative effect is 1.34 (95%CI 1.12–1.60).

**Table 1:** FDA definition of serious adverse event.^34^

Any adverse drug event occurring at any dose that results in any of the following outcomes:
1 Death
2 Life-threatening
3 Inpatient hospitalization (initial or prolonged)
4 Disability or permanent damage
5 Congenital anomaly/birth defect
6 Required Intervention to Prevent Permanent Impairment or Damage

The wealth of data available made it possible to verify that the adverse effects occur equally in the build-up and maintenance phases, nor can they be reduced by reducing the doses of immunotherapy administered. In synthesis, PACE showed that with high and moderate certainty evidence, POIT increases the chance and frequency of allergic reactions, anaphylaxis, use of epinephrine, and serious adverse events. While it is efficacious in increasing OFC thresholds, there is low certainty evidence that POIT may improve QoL compared to avoidance or placebo.

Shortly after the publication of PACE, a proprietary product used in two of the metanalyzed trials,^14^^,^^31^ AR101, underwent the Institute for Clinical and Economic Review (ICER) evaluation. This process is usual in the United States to evaluate the clinical and economic trade-offs of innovative prescription drugs. The ICER reports form the basis for establishing an appropriate “value-based price benchmark”, to be used by the policymakers. For AR101 (and for Viaskin Peanut), the ICER access and affordability alert indicated that the evidence is inadequate to demonstrate a health benefit compared to strict peanut avoidance. It defined the increase in the risk of allergic reactions and epinephrine use “expected, yet burdensome”. According to the reeport, there is not enough evidence to demonstrate that desensitization is effective in decreasing the reactions to accidental peanut exposure, nor to demonstrate an improvement of quality of life for peanut allergy sufferers.^36^ This did not prevent the FDA advisory committee on allergenic products from issuing a favorable judgment in its meeting on September 13, 2019.^37^

At this point, the discussion started to flare up. The points of the controversy are well summarized in a comment on the ICER judgment that appeared in November 2019.^38^ The points raised by this article are that ICER did not differentiate data on treatment-associated vs. accidental exposure-associated reactions to peanut, that ICER did not take into account that decreasing the risk of long-term or accidental reactions is an important factor for most patients, and that a different standard has been applied to peanut immunotherapy compared to immunotherapy for inhalant allergens or hymenoptera venom. In our opinion, reactions to peanut taken as immunotherapy are not more predictable and manageable than reactions during avoidance. Systemic reactions to subcutaneous immunotherapy (SCIT) in children are reported in 3.7–4.7% of cases,^39^^,^^40^ and in 0.07% using sublingual immunotherapy (SLIT).^41^ In POIT, 222 events per 1000 patients translate into a non-negligible 22%.

In synthesis, our opinion is that POIT should not escape the fundamental rule that a new therapy must commensurate the risks with the benefits. The necessity to abstain from doing harm is traced back to the time of Hippocrates and is effectively expressed with the Latin phrase “Primum, non nocere”.^42^ Useless for adults suffering from peanut allergy, POIT causes reactions very frequently also in children. The scope of this procedure is therefore limited to some specific situations, and cannot be proposed as the general solution for peanut allergy.

## The future

We see in the near future a series of necessities to ameliorate the care for peanut allergy sufferers—among them, safer schemes of POIT using the current approaches, the precise identification of the POIT-responder, the use of different peanut immunotherapies, and the use of biologics.

After the publication of the PACE meta-analysis, many other studies have already added to our knowledge. Among these, a new meta-analysis assessed 27 studies from the specific point of view of side effects.^43^ While it substantially confirms the PACE results, this study indicates some potentially useful “risk-reduction” avenues.

The risk of systemic reactions requiring epinephrine in this metanalysis was found at 7.6% (CI 4.5–11.4), higher in protocols including a rush phase (11.6%; CI 8.1–15.6%) and lower in studies with a cautious build-up phase (2.3%; 0.1–6.1%; p = 0.001). The authors found an increased risk of use of epinephrine in the protocols aiming at a high target maintenance dose (≥1000 mg: RR 13.7%; CI 9.6–18.3%) compared with protocols targeted at <1000 mg (RR 4.0%; CI 1.1–8.2%). Another risk factor for adverse events requiring treatment with epinephrine was a strong sensitization status, witnessed by high baseline peanut specific IgE (p = 0.0247) and larger baseline SPT wheal diameter (p = 0.0243). This metanalysis included also studies with POIT associated with the use of omalizumab, indicating that this is useful for a risk reduction. The “risk reduction” strategies suggested by the metanalysis include the use of omalizumab, cautious induction phases, low target doses, and the exclusion of patients with high sensitization rates. The main limitation of this meta-analysis is the inclusion of open-label and uncontrolled studies, reducing the quality of the evidence included in it. We are therefore rather perplexed about the possibility of reducing the side effects by maneuvering on the POIT technology.

**Fig. 1:**
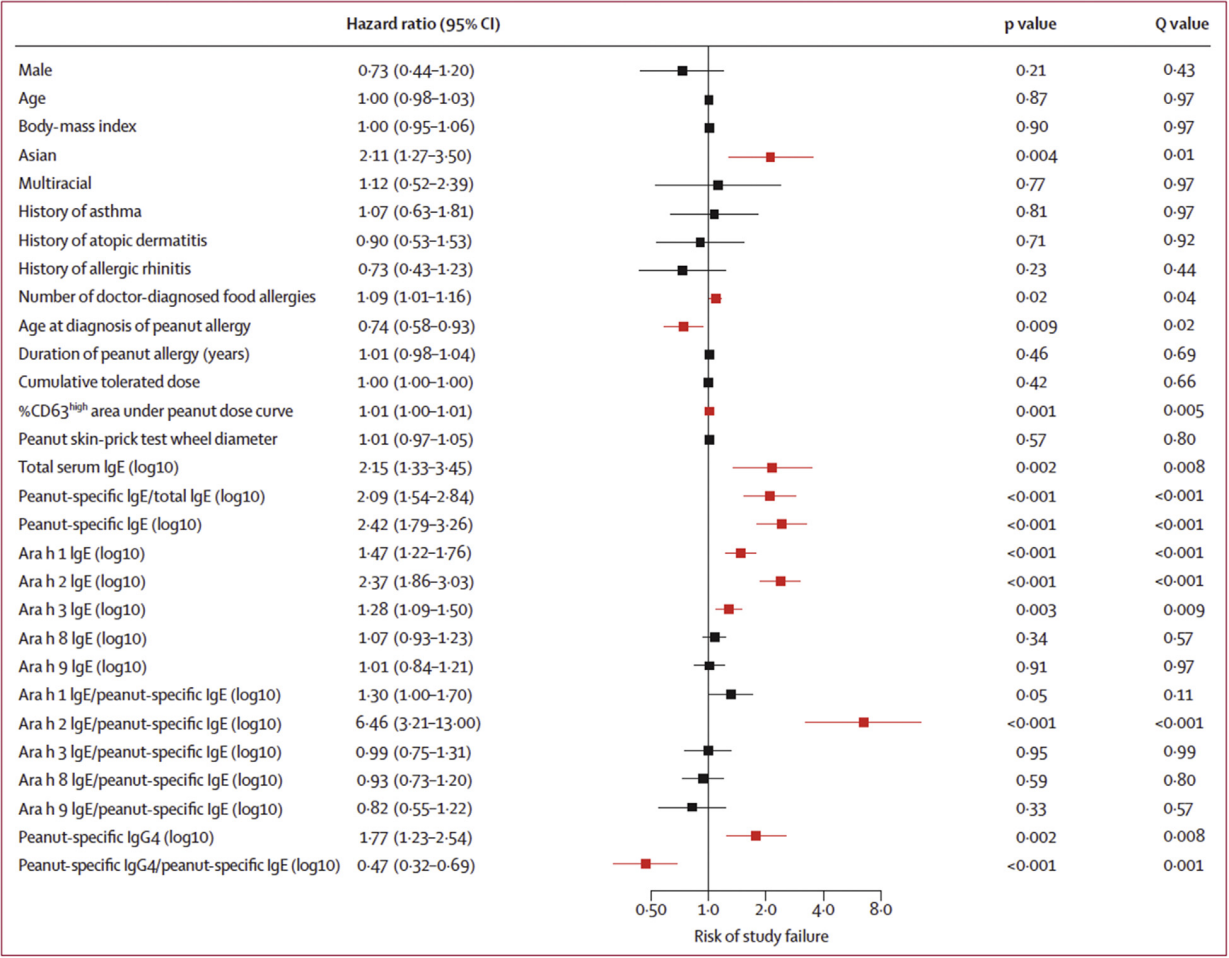
Hazard of study failure of POIT among baseline characteristics.^23^

The suggestion to treat children with POIT who do not present high risk factors for adverse reactions fits perfectly with the results of the POISED study.^23^ Among its findings, this study indicates some baseline risk factors for adverse events from POIT (high peanut-specific IgE/total IgE, high sensitization to Ara h 1 and Ara h 2, high basophil activation), and for lower odds of POIT success (high baseline peanut, Ara h 1, and Ara h 2 -specific IgE). In POISED, basal characteristics predictive of POIT success include high expression of CD63 at baseline, low Ara h 2 and peanut-specific IgE, high peanut specific IgG4. In short, as shown in Fig. 1, the child phenotype that will not respond fully to peanut-specific immunotherapy is characterized by high sensitization values.

If so, the ideal candidates for POIT are those with lower levels of sensitization, probably less risk of anaphylaxis, and higher reaction thresholds. Patients with high reactivity and anaphylactic reactions are sometimes excluded from the POIT protocols precisely because they are considered to be at excessive risk. Although food allergy phenotyping studies are scarce, this profile is similar to that of children at better prognostic indices of food allergy.^44^ Clearly, these are not those in greatest need of a solution to their problem. An ideal treatment for peanut allergy should target patients with high levels of antibodies, a low tolerance threshold, numerous episodes of accidental exposure even to minute quantities, and greater comorbidities. In the present forms, therefore, POIT is a good treatment for patients who need it least.^45^

One of the things that makes us hesitant in defining the ideal candidate for POIT is the lack of a univocal classification of severity of food allergy.^46^ We see the need to develop a form of classification of food allergies by severity, similar to that of asthma, based on how to decide in the future the opportunity of interventions with specific therapy, biologics,^47^ or simple food avoidance. In other words, we ask the scientific community to develop studies on the different phenotypes of peanut allergy.

A future scenario could see POIT becoming one of the options alongside safer forms of tolerance induction, such as epicutaneous immunotherapy,^48^ sublingual immunotherapy,^49^ or immunotherapy with inactivated allergens.^50^ Sublingual immunotherapy seems particularly well-tolerated, with 2% of doses eliciting mild reactions;^51^^,^^52^ its efficacy in desensitization is lower than OIT.^52^^,^^53^ On the model of immunotherapy for respiratory allergies, modified extracts are being studied as a possible safe and effective subcutaneous therapy for severely anaphylactic patients.^54^

The treatment possibilities are further widened with the entry of biologicals as partners or substitutes for POIT. The use of omalizumab in the treatment of peanut allergy has been proposed anecdotally for a long time.^55^^,^^56^ In multiple food allergy, it is able to get a substantial reduction of symptoms.^57^ A prospective evaluation of its efficacy in the treatment of multiple food allergy in peanut allergic children has been planned and is now ongoing. The ambitious OUtMATCH study^58^ is a three-stage project aimed to answer several practical questions of clinical importance. Among them, to which extent can omalizumab get peanut tolerance at double-blind placebo-controlled food challenge? Is the time of administration critical? In other words: does omalizumab, administered for a longer time, work better at decreasing allergic reactions? How does a short course of omalizumab combined with multi-allergen OIT compare with a longer course of omalizumab in decreasing allergic reactions? In other words: under treatment, will OIT be necessary, or is omalizumab sufficient? And finally, after participants stop both treatments, will they be able to eat the peanut and other foods in the form that is normally eaten? This ambitious project will pose the basis for more effective strategies in peanut allergy management.

## Conclusions

POIT studies have greatly contributed to shedding light on its possibilities and limitations, but POIT is not a panacea. Oral immunotherapy for peanuts is not for everyone and is not everything in the management of peanut allergy. The choice to undertake this route must be left to the case-by-case evaluation of the doctor and the patient's family. Probably the strategy we have already indicated 8 years ago regarding milk OIT also applies to POIT.^59^ In that review, we pointed out that clinicians and patients not ready to accept serious adverse effects from OIT would decide to continue the elimination diet; those motivated to achieving desensitization, and prepared to accept serious adverse effects, may choose to undergo immunotherapy. The same can happen with oral peanut immunotherapy. With the availability in the United States of the first standardized product useable for this purpose, together with the forthcoming availability of epicutaneous immunotherapies for the treatment of peanut allergy, a big help for sharing with the patient an appropriate decision may be the use of a specific shared decision-making tool.^60^

Alongside the traditional avoidance, very practicable in countries like Italy where the peanut does not have part of daily life, there are the possibilities of new forms of immunotherapy and biological therapies. While all these options are being explored, there is the need to identify the precise profile of the food-allergic child. The coming years will certainly see a rapid evolution in this field, and we will be able to arrive at a sartorial choice for each situation.

## Author contributions and consent for publication

The authors collaborated in equal degree to the conceptual elaboration of the text. They express their consent to publication.

## Funding

No funding specifically secured for this article.

## Ethical approval

As the text does not contain any evaluation of data directly drawn from patient caseloads but only a reasoned review of the literature, it is not subject to ethical approval.

## Availability of data and materials

All the materials used for this review are published.

## Declaration of Competing Interest

Dr. Fiocchi reports currently sponsored research by 10.13039/100007773Danone/Nutricia, the Netherlands, 10.13039/100004339Sanofi/Regeneron, U.S.A., Hipp, Germany, Ferrero, Italy. He is on advisory boards of Danone, Stallergenes, France, Menarini, Italy, Abbott, U.S.A., DBV, U.S.A. - France, Novartis, Switzerland, and Hipp. Dr. Mennini is on advisory board of Biogaia, Sweden.
